# Is YouTube a Reliable Source for Laryngomalacia Information?

**DOI:** 10.7759/cureus.85252

**Published:** 2025-06-02

**Authors:** Jeremy Walsh, Lauren A DiNardo, Austin Knorz, Emilie Christie, Alyssa D Reese, Kristina Powers, Michele M Carr

**Affiliations:** 1 Surgery, Upstate University Hospital, Syracuse, USA; 2 Otolaryngology, University of Rochester Medical Center, Rochester, USA; 3 Anesthesiology, University at Buffalo Jacobs School of Medicine and Biomedical Sciences, Buffalo, USA; 4 Surgery, Northwell Health, New York, USA; 5 Plastic and Reconstructive Surgery, Eastern Virginia School of Medicine, Norfolk, USA; 6 Otolaryngology, Eastern Virginia School of Medicine, Norfolk, USA; 7 Otolaryngology, University at Buffalo Jacobs School of Medicine and Biomedical Sciences, Buffalo, USA

**Keywords:** discern score, laryngomalacia, online health information-seeking, supraglottoplasty, youtube

## Abstract

Objective

The goal of our study was to assess information pertaining to laryngomalacia on YouTube.

Methods

On February 6, 2022, YouTube (www.youtube.com) was searched for "laryngomalacia," and the first 100 videos based on "relevance" were included. Videos were excluded if they did not discuss laryngomalacia or if the audio was not in English. Two medical students independently viewed and analyzed each video. Each video was evaluated for author, category, goal, video quality, audio quality, and basic YouTube metrics. Videos were also evaluated for specific information about laryngomalacia, including a definition of laryngomalacia, how it is diagnosed, symptoms mentioned, and treatment options. A modified DISCERN criteria score was included to evaluate the quality of information in each video.

Results

Ninety-five videos were included. The most common video authors were patient caregivers (N = 33, 34.7%). The goal of 31.6% (N = 30) of the videos was to demonstrate the symptoms of laryngomalacia, while 25.3% (N = 24) provided a complete overview of laryngomalacia. Videos made for educational purposes by hospitals and providers were statistically more likely to be of better quality regarding video clarity, text, and graphics (p = 0.001) when compared to videos classified as testimonials and patient caregiver experiences. Moreover, 48.3% (N = 28) of the educational videos were classified as 3/3 for video quality, while 10.8% (N = 4) of the testimonials and patient caregiver experience videos were rated as 3/3. The majority of the videos met only one of the five DISCERN criteria (N = 56, 58.9%). Only one of the videos (N = 1, 1.0%) shared resources with viewers.

Conclusion

The stridor that often accompanies laryngomalacia can be concerning for parents to witness in their child. When parents and caregivers search for information about laryngomalacia on YouTube, they typically find home videos focused on testimonials and personal caregiver experiences. Referring parents to specific, high-quality social media resources is crucial to reduce misconceptions given the wide variety of presentation and severity of laryngomalacia.

## Introduction

The rise of digital medical resources and social media platforms has empowered patients with greater access to information regarding their health conditions, encompassing symptoms, diagnosis, prognosis, and treatment options. Over 65% of adults rely on the Internet for medical information [1]. Moreover, with approximately two billion people accessing YouTube daily, the platform has emerged as a significant source of non-peer-reviewed medical information [2]. Consequently, physicians are becoming increasingly aware of YouTube's impact on patient education [3].

Previous research has found that many parents with otolaryngology questions and concerns turn to the Internet for information on their child's condition [4]. The Health Information National Trends Survey (HINTS) reported that 80% of Internet users access health information online [3]. However, with the vast resources available online, it can be difficult for patients to determine the credibility of the information. Thus, there have been numerous studies looking at the quality of health-related information on YouTube in a multitude of specialties, including otolaryngology [2-11].

Laryngomalacia is a common congenital anomaly of the larynx of infants, characterized by the collapse of soft tissues above the vocal cords during inspiration, resulting in stridor [12]. Most commonly, infants present with stridor and feeding-related symptoms [13]. Severe cases can lead to failure to thrive, weight loss, cyanosis, pulmonary hypertension, and other sequelae of airway obstruction [13,14]. The severity of the symptoms correlates with the degree of intervention required, ranging from medical management to surgery [13]. Supraglottoplasty is the mainstay surgical intervention for laryngomalacia. This procedure involves the removal of excessive supraglottic tissue and relaxation of the aryepiglottic folds via the cold technique or laser, usually CO2 [15,16]. 

The symptoms of stridor and respiratory distress can often cause stress in family members, causing them to seek further information, often on the Internet. Corredera et al. previously looked at online information on laryngomalacia and found variable quality [17]. However, YouTube is a unique source to look at, given that laryngomalacia can cause noisy breathing, and this noise can be heard in the video format. Moreover, laryngomalacia presents in newborn infants, causing stress in young adults who are intimately connected to social media, including YouTube. Our study aims to evaluate the quality of information on YouTube about laryngomalacia. We hypothesize that the videos on YouTube pertaining to laryngomalacia are unreliable and inaccurate.

## Materials and methods

On February 6, 2022, a YouTube search was performed from a new account using the search term “laryngomalacia.” A second, independent researcher performed the search again on a different device to ensure search results were comparable. The first 100 videos based on “relevance” were included to replicate the average search attempt. Videos were excluded if they did not discuss laryngomalacia or if the audio was not in English.

Reviewers were medical students who had attended at least one lecture on laryngomalacia aimed specifically at the key features included in the video analysis. Two independent reviewers evaluated the videos for their eligibility. Any discrepancies were discussed between the two reviewers and resolved by a third independent reviewer. 

Basic YouTube metrics were recorded for each video, including the video link, video title, duration, views, upload date, likes, and comments. We utilized the modified DISCERN criteria, initially defined by Singh et al. [6] to assess the quality and reliability of the videos. The modified DISCERN criteria were adapted from the original DISCERN criteria defined by Charnock et al. [18], which assessed the quality of written health information for consumers. The modified DISCERN criteria have been adapted to assess the quality of information in video format. The modified DISCERN criteria assess clarity, bias, reliability, reference supplementation, and areas of uncertainty [6]. Each criterion gets one point if it is met, and a score of five total points reflects the highest level of reliability. The Kappa score is 0.53 and improves with practice [18]. Author types were categorized into US otolaryngologist, non-US otolaryngologist, US physician (non-otolaryngologist), non-US physician (non-otolaryngologists), YouTube influencer (non-health professional, not a patient's family or friend), non-MD professional, patient’s friends and family, unknown, hospitals, or companies/advertisers. The videos were categorized as either educational or testimonial/patient’s caregiver experience. The goals of the videos were categorized based on the main topic of the video, including what is laryngomalacia, patient’s caregiver experience with laryngomalacia, recognizing the signs and symptoms, treatment and management at home, how to diagnose laryngomalacia, complete overview, or other. Video and audio quality were rated on a scale of 1 to 3. Lastly, we assessed whether the videos addressed specific valuable questions pertaining to laryngomalacia, like “what is laryngomalacia?”, “how is the diagnosis made?”, “were common signs and symptoms addressed?”, “how is laryngomalacia treated?”, and “did they describe supraglottoplasty?”. Using the DISCERN criteria and the secondary video characteristics, we developed a system to evaluate the quality and validity of the videos on YouTube regarding laryngomalacia.

Statistical analysis

Statistical analysis was performed using IBM SPSS Statistics for Windows, Version 25.0 (released 2017, IBM Corp., Armonk, NY). Inter-rater agreement was assessed through the utilization of the intraclass correlation coefficient (ICC) and its corresponding 95% confidence intervals (CIs). The analysis was based on a mean rating (k = 2), using a two-way random model to account for consistency. Descriptive statistics, including measurements such as mean, standard deviation, median, frequency, and percentage, were used to assess the data collected. Categorical variables were represented using frequencies, while continuous variables were presented using means and standard deviations. The Chi-square test was used to assess correlations between categorical variables. A p-value <0.05 was considered statistically significant.

## Results

Of the 100 videos initially identified from the YouTube search, 95 videos were included based on our criteria. Video viewership ranged from five to 427,016 views, with a mean of 20,088.2 views (SD = 51,447.4). Days available varied from 130 days to 4,703 days with a mean of 1680.3 days (SD = 1332.1). The number of likes on each video varied from 0 to 6,695, with a mean of 170.8 likes (SD = 776.6); the number of comments found for each video ranged from 0 to 238, with a mean of 11.3 comments (SD = 32.2). Only one video included references, resulting in 98.9% of videos excluding any references supporting the information posted.

The authors of the videos were most often a patient’s family or friend (N = 33, 34.7%), followed by unknown (N = 20, 21%) and non-US otolaryngologists (N = 12, 12.6%) (see Table 1). The category of the video was typically educational in nature (N = 58, 61%) (Table 1). The goal of 31.6% of the videos was to demonstrate the signs and symptoms of laryngomalacia, followed by 25.3% of videos attempting a complete overview of the condition (Table 1). The mean score for video and audio quality was 2.31 (SD = 0.53) and 2.48 (SD = 0.73), on a three-point scale, respectively (Table 2). When addressing the specific questions pertaining to laryngomalacia, 74.7% of videos addressed the signs and symptoms of laryngomalacia. Less than half defined laryngomalacia (46.3%), and only 26.3% described what supraglottoplasty was (Table 2). When correlating specific topic questions to author type, the patient's family and friends discussed the signs and symptoms of laryngomalacia in 100% of their videos (N = 33), with the next closest specific question addressed at 21.2%. Otolaryngologists (US and non-US) were more likely to define laryngomalacia compared to the rest of the author types combined (68.4% vs. 40.8%, respectively, OR = 3.15, 95% CI = 1.08-9.17, p < 0.05). 

**Table 1:** Author types, category of video, and goals of video (N = 95)

Author type	N (%)
Patients’ family or friends	33 (34.7)
Unknown	20 (20.1)
Non-US otolaryngologist	12 (12.6)
Corporate advertising	9 (9.5)
Non-otolaryngologist non-US MD	7 (7.4)
US-otolaryngologist	7 (7.4)
Hospital	2 (2.1)
YouTube influencer	2 (2.1)
Non-otolaryngologist US MD	2 (2.1)
Non-MD professional	1 (1.1)
Category of video	
Educational	58 (61)
Testimonial	37 (39)
Goal of video	
Recognition of symptoms	30 (31.6)
Comprehensive overview	24 (25.3)
Describing laryngomalacia	17 (17.9)
Treatment/management at home	13 (13.7)
Parent's experience of the child with the condition	5 (5.3)
Other	4 (4.2)
Diagnosing laryngomalacia	2 (2.1)

**Table 2:** Video quality, audio quality, and topic-specific questions (N = 95)

Video quality	Description	N (%)
Poor	Visuals are blurry, grainy, or difficult to understand	3 (3.2)
Fair	Regular video quality, average text clarity	60 (63.2)
Good	Clear visuals and text with some professional graphics or effects	32 (33.7)
Audio quality	Description	
Poor	No audio	13 (13.7)
Fair	Speech was difficult to understand, or had background noise	23 (24.2)
Good	No difficulty understanding spoken words	59 (62.1)
Topic-specific questions	Description	
Signs/symptoms of laryngomalacia	Description of stridor, retractions, feeding problems, etc	71 (74.7)
What is laryngomalacia	States that it is due to laryngeal collapse	44 (46.3)
How is laryngomalacia treated?	Reflux management, positioning, monitoring, or surgery	33 (34.7)
How is the diagnosis made?	States the use of flexible laryngoscope	30 (31.6)
Described supraglottoplasty	Describes the procedure with some detail	25 (26.3)

The DISCERN scores ranged from 0 to 4, with a mean of 1.54 (SD = 0.99). No video was able to get all five points in the DISCERN criteria. The majority of the videos were graded as 1 (N = 56, 58.9%), followed by 2 (N = 19, 20%), 3 (N = 8, 8.4%), 4 (N = 7, 7.4%), and 0 (N = 5, 5.3%) (Table 3). DISCERN scores were found to be significantly correlated with author type (Table 4). Otolaryngologists were found to have statistically significantly higher DISCERN scores compared to friends and family (p < 0.05). Scoring for otolaryngologists revealed 42.1% receiving either a 3 or 4 on the modified DISCERN criteria, while only 15.8% scored a 0 or 1. Comparatively, 97% of videos by friends and family scored a 1 on the modified DISCERN criteria, with zero videos exceeding a score of 2 (Table 4). When comparing the number of views a video received to the DISCERN score, there was no statistical significance found between groups. Furthermore, there was no significant difference in the mean number of views of videos created by otolaryngologists and those produced by friends and family. 

**Table 3:** Modified DISCERN score of videos meeting criteria (N = 95)

DISCERN score	N (%)
0	5 (5.3)
1	56 (58.9)
2	19 (20.0)
3	8 (8.4)
4	7 (7.4)
5	0 (0.0)

**Table 4:** Modified DISCERN scores of different author types Pearson Chi-square X^2^ = 43.373, df = 8, p-value < 0.01. * The maximum DISCERN score is 5.

Modified DISCERN score	0	1	2	3	4		
Author type						Total (N = 95)	Mean*
US ENT	0	0	2	4	1	7	2.86
Non-US ENT	1	2	6	1	2	12	2.08
Non-ENT US MD	0	0	1	0	1	2	3.00
Non-ENT Non-US MD	0	5	1	0	1	7	1.57
YouTube influencer	0	2	0	0	0	2	1.00
Non-MD professional	0	0	1	0	0	1	2.00
Patient’s family and friends	0	32	1	0	0	33	1.03
Unknown	4	12	3	1	0	20	1.05
Hospital	0	1	0	1	0	2	2.00
Corporate advertising	0	2	4	1	2	9	2.33

## Discussion

Since its inception, many have used YouTube to learn about health conditions [1]. However, this accessibility raises concerns about the accuracy and reliability of information, as reported in the literature [2-6,8-9]. As we hypothesized, the videos about laryngomalacia on YouTube were found to be unreliable, evident in our low total DISCERN score. However, the DISCERN scores for US and non-US otolaryngologists were statistically higher compared to the mean of our cohort (2.87 and 2.08 vs. 1.54, p < 0.05, respectively), yet there was no statistical difference between the number of views for each DISCERN score, meaning that the video quality did not influence views. This discrepancy emphasizes the challenges of ensuring the credibility of medical information on YouTube and warrants further examination into the factors that influence video reliability.

Once Keelan et al. [9] revealed how YouTube videos can be misleading and promote distrust in vaccine science, many others have taken an interest in identifying the accuracy of YouTube videos in their respective fields [2-6,8-9]. Wu et al. [5] demonstrated the variability and unreliability of YouTube videos about functional endoscopic sinus surgery. Like our study, theirs reported a higher DISCERN score for universities/professional organization videos (DISCERN score of 2.37 + 1.25) compared to those posted by medical advertisers/for-profit companies (DISCERN score of 1.46 + 1.17), suggesting that universities and professional organizations present information with less bias [5]. Similarly, Ward et al. [10] concluded that videos by US board-certified physicians had an overall mean DISCERN score significantly higher (p < 0.001) than other author types (3.00 vs. 2.38, respectively) when evaluating the quality of information for common pediatric otolaryngology procedures. Wu et al. [5] concluded that there was a lack of correlation between higher DISCERN scores and video engagement. The overall theme of these studies parallels the results and concerns described in our study, implying that reliable, high-quality videos are not being given priority over poor-quality videos.

Healthcare workers are starting to see the impact of YouTube videos as they shape patients’ perception and understanding of their medical condition. For example, Sorensen et al. [4] illustrated how a news program, while discussing a child who suffered a fatal postoperative hemorrhage following a tonsillectomy, inadvertently overemphasized the significance of this rare outcome. Such videos frequently contribute to bias and omission of comprehensive information [4]. Similarly, our study showed that the majority of videos about laryngomalacia addressed symptoms of the disease (74.7%). Although videos pertaining to signs and symptoms of laryngomalacia may encourage families to seek a clinical diagnosis, these videos provide little to negligible value beyond that. This can cause patients to overlook videos that may offer better educational value, as exemplified by our study, which found that only a fraction of videos explained what laryngomalacia is, treatment/ management, and how to diagnose laryngomalacia. These findings are problematic as these videos are inherently more valuable to the patient’s understanding of the condition, but account for a minority of videos. 

While questioning the credibility of YouTube videos, one might ask if the videos are more harmful than beneficial. Radonjic et al. [2] researched the credibility of information about abdominal aortic aneurysms on YouTube and concluded that the majority of videos were either very poor (31%) or poor (41%) with regard to accuracy and reliability. Similar to Wu et al. [5] and our study, Radonjic et al. [2] found videos produced by nonphysicians and patients were most popular but had the lowest reliability scores. Haymes et al. [7] found similar results assessing information quality on YouTube pertaining to epistaxis. They found that only half of the videos provided proper epistaxis guidance, such as leaning forward, where to apply pressure, and for how long to hold pressure [7]. Even more concerning, 47% of videos contained inappropriate advice, such as using a tampon intranasally or rubbing black pepper on the septum, to name a few [7]. Due to the variability of the videos and inappropriate medical advice, Haymes et al. concluded that YouTube should not be a source of medical information [7].

The results of this study carry implications for patients, parents, and physicians. While there are credible and resourceful videos, there is no correlation between credibility and views. Osman et al. [10] implied that YouTube's popularity-based system may contribute to the disconnect. Thus, educational videos produced by universities, which are superior in quality, accuracy, and content, are being viewed less frequently than untrustworthy videos [11]. To counteract this problem, healthcare professionals can recommend credible and educational videos to improve patients' knowledge of diseases.

This study has potential limitations. Videos were chosen based on the YouTube algorithm, so relevant videos may have been missed. Non-English videos were excluded, limiting worldwide generalizability. There is no standard protocol to determine the accuracy and reliability of videos pertaining to laryngomalacia. Therefore, we created our own tool to assess the videos, based on whether specific questions about the topic were addressed; this was conducted with the help of a pediatric otolaryngologist and relevant literature [12-16]. In addition, our data were a result of a cross-sectional study, which only examined YouTube videos at a single time point. Considering the vast volume of content added to the platform every day, it is possible that newer and more relevant videos will become available in the future. Moreover, we did not search any other video-based platform due to the popularity and accessibility of YouTube. Comparison groups based on creator category were small, which limited the power to detect differences between the groups. Lastly, the analysis of the videos was conducted by medical students who were instructed on the topic of laryngomalacia but are not experts in the field.

## Conclusions

The widespread use of YouTube for health information related to laryngomalacia raises concerns about the accuracy and reliability of its content. Videos created by otolaryngologists were significantly more accurate and reliable; however, these were often overshadowed by the abundance of low-quality content, as demonstrated by the lack of a correlation between video quality and viewership. A multifaceted strategy is needed to improve the visibility and credibility of trustworthy medical videos on the platform. This includes high-quality, targeted, and clinician-produced videos on laryngomalacia and other pediatric airway conditions that can be distributed to patients and their families. Collaboration between medical societies and digital platforms could potentially improve the credibility and quality of content through algorithmic prioritization, verified physician labels, and content endorsement.
